# Microscopic Distribution of Quaternary Ammonium Salt Organic Modifiers in the Interlayer Space of Montmorillonite: Molecular Simulation Study

**DOI:** 10.3390/ma18102338

**Published:** 2025-05-17

**Authors:** Wenxi Yu, Xijian Yi, Jianwei Yan, Juan Cheng, Siyu Ou, Qiong Xue

**Affiliations:** 1 School of Packaging Engineering, Hunan University of Technology, Zhuzhou 412007, China; yuwx@hut.edu.cn (W.Y.); m22085600024@stu.hut.edu.cn (X.Y.); m24080500009@stu.hut.edu.cn (S.O.); xueqiong@hut.edu.cn (Q.X.); 2 State Key Laboratory of Performance Monitoring and Protecting of Rail Transit Infrastructure, East China Jiaotong University, Nanchang 330013, China; 3 College of Packaging Engineering, Jinan University, Zhuhai 519070, China; chengj2008@jnu.edu.cn

**Keywords:** montmorillonite, organic modification, quaternary ammonium salts, molecular dynamics simulation

## Abstract

This study employs molecular dynamics simulations to construct designed unit cells of organic montmorillonite (OMMT) modified with four types of quaternary ammonium salts. The effects of modifier type and quantity on the basal spacing of montmorillonite (MMT) were analyzed. Molecular motion, morphology, interaction energy (*E*_int_), and hydrogen bonding interactions were investigated to elucidate the molecular-level mechanisms between modifiers and MMT. The results indicate that the organic modification of MMT proceeds in three distinct stages: the filled stage, saturated stage, and supersaturated stage. During the filled stage, the basal spacing remains largely unchanged while *E*_int_ increases rapidly. In the saturated stage, the basal spacing expands as the growth rate of *E*_int_ slows. In the supersaturated stage, the basal spacing continues to increase while *E*_int_ stabilizes. The transition from the filled to saturated stage is governed by the van der Waals space occupied by the modifiers. Within the MMT interlayer, the modifiers adopt a bilayer morphology, with the nitrogen atom heads adhering to the MMT surfaces and the tails self-assembling. These findings provide theoretical insights into the basal spacing expansion and organic modification mechanisms of MMT, thereby facilitating improved material compatibility.

## 1. Introduction

Montmorillonite (MMT) is a natural hydrated aluminosilicate mineral that has garnered increasing attention due to its abundant availability, low cost, and environmental friendliness. It finds extensive applications in various fields including electrochemistry [1], catalysis [2,3], adsorption [4,5], antibacterial applications [6,7], medical applications [8,9], and nanocomposites [10,11]. The structural unit layer of MMT consists of two silicon-oxygen tetrahedral sheets sandwiching an aluminum–oxygen octahedral sheet [12]. Its residual layer charge originates from the partial substitution of Si^4+^ by Al^3+^ in the tetrahedral sheets and the partial substitution of Al^3+^ by Mg^2+^ in the octahedral sheets [13]. The layered structure of MMT exhibits hydrophilic and lipophobic characteristics, leading to poor compatibility with organic materials [14]. Furthermore, the stacked lamellar configuration significantly reduces its dispersibility, thereby limiting its applications in polymer packaging materials. The key challenges for realizing its commercial value lie in expanding the basal spacing of MMT and enhancing its compatibility with polymeric materials.

The lamellar stacking of MMT primarily relies on weak electrostatic interactions and van der Waals forces, a characteristic facilitating cation exchange within its interlayers [15]. Among various modification approaches for MMT, cationic organic modifiers have attracted significant research attention. Li et al. [16] synthesized organically modified montmorillonite (OMMT) with expanded basal spacing using dodecyl dimethyl betaine, which demonstrated substantially enhanced adsorption capacity for lead ions. Chen et al. [17] achieved homogeneous dispersion of OMMT in bismaleimide composites, thereby markedly improving the mechanical properties of the materials. Kodali et al. [18] employed quaternary ammonium salt modifiers to prepare OMMT and its composites, revealing that modifier incorporation increased MMT’s basal spacing, promoted uniform distribution in the matrix, and significantly enhanced composite thermal stability. Organic modifiers effectively expand MMT’s basal spacing while reducing interlayer polarity, thereby improving dispersibility and interfacial compatibility with other materials. These enhanced properties collectively elevate the overall performance of MMT-based composites [19], substantially broadening their application potential across multiple fields.

Current investigations have predominantly examined the modification efficacy of modifiers on MMT or the impacts of OMMT on composite materials. However, due to limitations in experimental methodologies, the microscopic mechanisms underlying MMT–modifier interactions at the molecular level remain difficult to characterize qualitatively or quantitatively. Molecular dynamics (MD) simulations, capable of revealing microscopic interaction mechanisms between materials at the molecular level, have been extensively applied to investigate MMT systems. Yang et al. [20] investigated the dynamic wetting process of MMT/H2O systems using MD simulations. Zhang et al. [21] explored the influence patterns of substance transport and mechanical weakening characteristics during MMT hydration under varying moisture content, temperature, and pressure conditions through MD simulations. Gao et al. [22] examined the effects of layer charge density on MMT exfoliation processes and the surface electrical properties of exfoliated nanosheets via MD simulations. In these investigations, partial atoms of MMT were fixed at spatial coordinates within the lattice cell, restricting MMT layers to positional changes solely through overall lattice cell expansion/contraction, while the basal spacing of MMT depended on initial configurations. MMT cannot be treated as a discrete molecule, a constraint that may prove advantageous for investigating MMT layer surface properties. Nevertheless, the lattice cell model fails to provide adequate spatial accommodation for molecular chain segment mobility in long-chain molecules such as quaternary ammonium modifiers. In this study, we first constructed an MMT lattice cell model and subsequently eliminated its three-dimensional periodicity to generate an isolated molecular structure. The independent MMT molecule was then incorporated into the designed unit cell model for advanced investigations, with the designed unit cell model providing sufficient spatial dimensions to support unrestricted movement of molecular chains during model construction. Furthermore, the unit cell model enables systematic investigation of MMT’s holistic transformations, thereby facilitating the exploration of molecular motion in MMT and its interaction patterns with other substances.

The objective of this study is to investigate the influence of four organic modifiers (hereinafter referred to as modifiers), including octadecyl trimethyl ammonium chloride (OTAC), cetyl trimethyl ammonium chloride (CTAC), octadecyl dimethyl benzyl ammonium chloride (ODBA), and dioctadecyl dimethyl ammonium chloride (DDAC), on the basal spacing of OMMT with varying substitution amounts through MD simulations. These four organic modifiers, serving as prevalent agents in MMT modification, provide well-defined structural variations (C_16_–C_36_ alkyl chains, aromatic/linear configurations) that facilitate systematic exploration of architectural effects on MMT. The results identified three characteristic stages during MMT organic modification, specifically the filled stage, the saturated stage, and the supersaturated stage. The MMT–modifier interactions were further elucidated at the molecular scale through combined analysis of molecular motion and morphology, interaction energy (*E*_int_) calculations, and hydrogen bond (H-bond) characterization, providing mechanistic insights unattainable through experimental measurements alone. The insights will ultimately contribute to the development of modification strategies for optimizing MMT compatibility with diverse materials and enhancing its applicability.

## 2. Computational Details

### 2.1. Computational Method

In this work, MD simulations were conducted to study MMT and PVA using the Forcite module and Amorphous Cell module of Material Studio. In all simulations, the Ewald summation method was adopted for the electrostatic energy part of non-bonded energy, and atom-based summation was applied for van der Waals energy calculations, with a cut-off distance of 12.5 Å [23]. The Interface force field was chosen to describe the system. This force field has demonstrated efficacy in simulating both inorganic–organic and inorganic–biomolecular interfacial systems [24], showing notable applicability for modeling MMT and PVA materials.

Geometric optimizations were executed using the Smart-algorithm, which is an iterative protocol integrating Steepest Descent, Adjusted basis set Newton–Raphson, and Quasi–Newton methodologies [25]. A time step of 1 fs was employed [26], with its maximum allowable value dictated by the most rapidly varying forces within the system [27].

### 2.2. Molecular Models

The molecular models of four modifiers (OTAC, CTAC, DDAC, and ODBA) were established based on their structural formulas, with hydrogen atoms (H) depicted in white, carbon atoms (C) in gray, oxygen atoms (O) in red, and nitrogen atoms (N) in blue. The validated MMT model was derived from Heinz [28] under the Interface force field framework. After eliminating the periodic arrangement of the MMT crystal lattice, two parallel-aligned MMT layers were constructed by introducing 20 potassium ions (K^+^) to maintain charge neutrality. In the MMT model, purple represents potassium, yellow represents silicon, pink represents aluminum, and green represents magnesium. The cation exchange capacity of this MMT model is 100 meq/100 g, consistent with typical MMT materials [29]. The basic information is summarized in Table 1. Geometric optimization was performed over 10,000 steps.

A three-dimensional periodic unit cell of (1 P1) type constructed lattice parameters of *a* = *b* = 28 Å and *c* = 80 Å. Two MMT layers were positioned at opposite ends of the unit cell, followed by the insertion of one OTAC molecule at the geometric center after the removal of one K^+^, thereby designating this configuration as OTAC1. In an analogous arrangement, two OTAC molecules were centrally positioned following the removal of two K^+^, resulting in the OTAC2 unit cell. Using an analogous methodology, the substitution amounts of the modifiers were systematically varied from 1 to 20, with 80 corresponding unit cells constructed.

### 2.3. Molecular Dynamics Simulations

**Figure 1:**
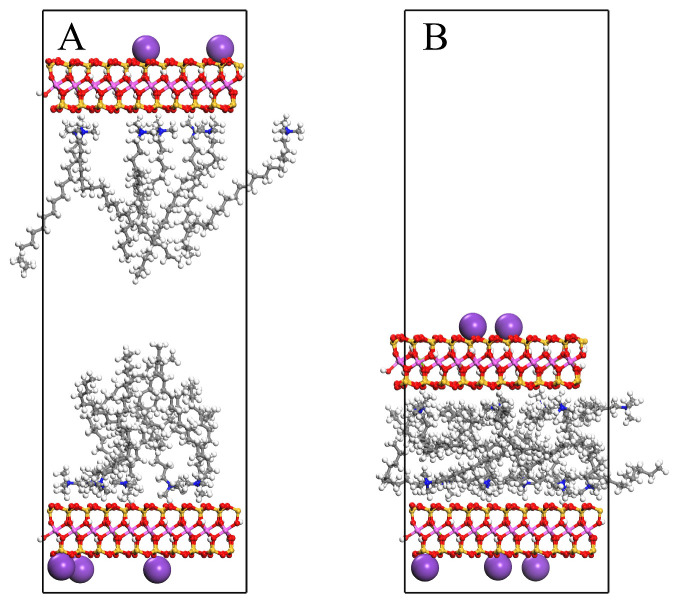
The OTAC15 unit cell: (**A**) before MD simulation; (**B**) after MD simulation.

The same geometric optimization was performed on the unit cells to minimize the possibility of atomic overlap during modifier intercalation. Subsequently, the upper MMT layer was subjected to a 10 kcal·mol^−1^·Å^−1^ force along the (*X* = 0, *Y* = 0, *Z* = 1) direction during MD simulations. Simulations were conducted under the canonical ensemble (NVT) for 2000 ps. Figure 1 illustrates the molecular morphology of the unit cell before and after MD simulation under an applied force, using OTAC15 as a representative example. Under the applied force, the basal spacing of MMT gradually decreased as the simulation progressed. During the application of force, the modifiers had sufficient space for movement.

**Figure 2:**
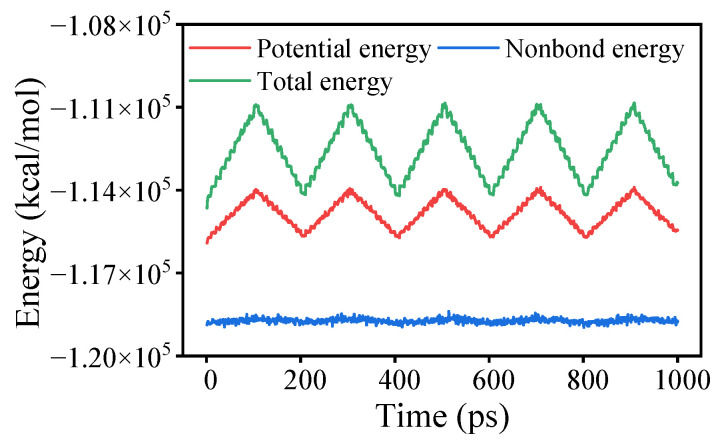
Energy variations in the OTAC15 unit cell in anneal.

Annealing using the NVT ensemble was conducted following the force-applied MD simulation. The annealing process comprised 5 cycles with temperatures ranging from 298 K to 598 K. As a computational technique, annealing systematically combines geometric optimization and MD simulations through controlled temperature variations. This method employs a stochastic optimization algorithm to achieve iterative convergence toward optimal configurations, effectively reducing internal stresses generated during model construction while minimizing unit cell energy, thereby enhancing structural stability. Figure 2 illustrates the energy changes during the annealing process, using OTAC15 as an example. Subsequently, a 1000 ps MD simulation was performed on the unit cells under the NVT ensemble at 298 K. Data analysis was conducted based on the files generated from this simulation step.

## 3. Results and Discussion

### 3.1. Stability of the Model

**Figure 3:**
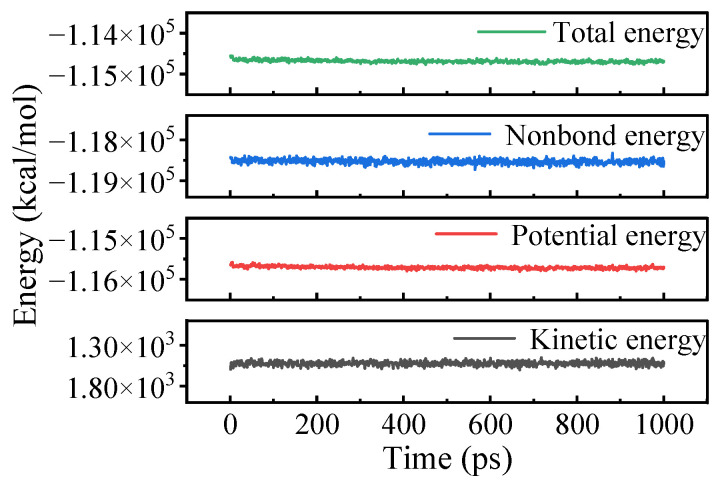
Energy variations in the OTAC15 unit cell in MD simulation.

The unit cell must be equilibrated before conducting MD simulations. Figure 3 illustrates the energy variation over time during the NVT MD simulations, using OTAC15 as an example. The system energy remained stable with fluctuations within 5%, demonstrating successful equilibration. Comparable energy convergence patterns were observed across all unit cell configurations, validating the reliability of subsequent analysis.

### 3.2. Basal Spacing

**Figure 4:**
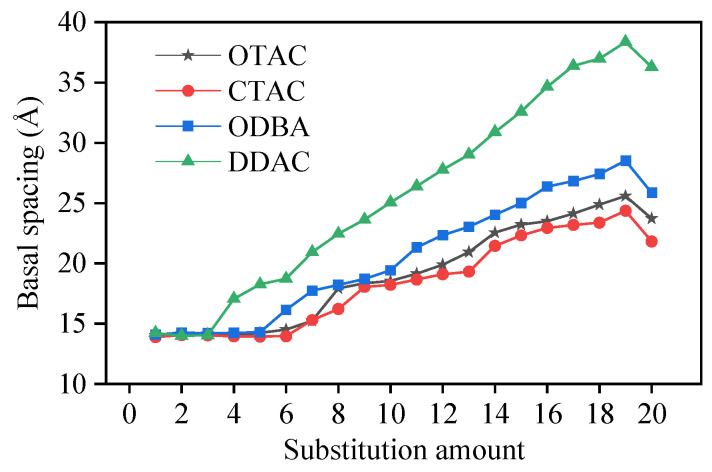
The variation in base spacing with different substitution amounts.

The incorporation of modifiers increases the basal spacing (d_001_) of MMT [17], facilitates interlayer separation, and improves the dispersion of MMT. The basal spacing of MMT was determined by measuring the distance between Miller planes. Figure 4 illustrates the correlation between substitution amounts and basal spacing.

In our previous study [30], four OMMT variants with a substitution degree of 2 exhibited basal spacings of 13.42–15.28 Å within the PVA matrix. Narrower basal spacing ranges of 13.99–14.25 Å with elevated mean values were demonstrated by these OMMTs under identical substitution conditions in this work. This discrepancy originates from the higher PVA matrix density in prior experiments compared to the vacuum environment employed here, where stronger PVA–MMT interfacial interactions restricted interlayer expansion. The MMT basal spacing was determined as 12.30 Å through macroscopic experimental characterization [31]. The simulation–experiment discrepancy originates from the absence of water molecules in unit cells. Independent modeling produced a 12.29 Å basal spacing for hydrated MMT, consistent with experimental measurements and validating the unit cell’s structural fidelity.

**Figure 5:**
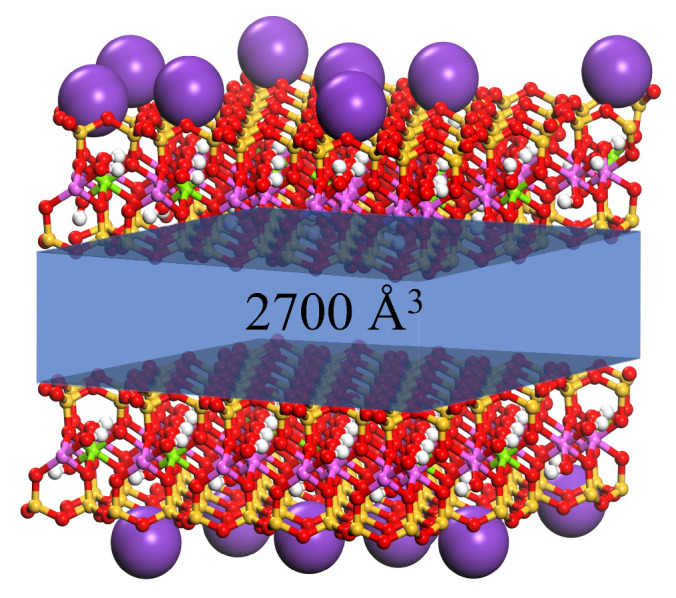
Schematic diagram of the interlayer space volume in MMTs.

The simulated results demonstrated that basal spacing remained stable at low substitution amounts, began to progressively expand upon reaching a critical substitution threshold, and exhibited stepwise enlargement with further substitution increases. The basal spacing of MMT exhibited a relative decrease upon increasing the substitution amount of modifier molecules from 19 to 20. This phenomenon originated from K^+^ displacement around MMT layers triggered by complete substitution, which drove the system’s transition from a three-component (MMT, modifier molecules, K^+^) to a two-component (MMT, modifier molecules) configuration. Within the unit cell, K^+^ maintained charge balance and provided external interaction forces to MMT, leading to reduced basal spacing after complete K^+^ removal. The substitution amount nodes (5 for OTAC, 6 for CTAC, 5 for ODBA, 3 for DDAC) mark the transition threshold from stable to expanding basal spacing. The van der Waals volume occupied by the modifier molecules determines the magnitude of the substitution amount nodes. Simulation measurements identified 2700 Å^3^ (Figure 5, blue region) as the threshold interlayer volume required to trigger MMT basal spacing expansion.

Among different modifiers under identical substitution amounts, the basal spacing decreases in the order of DDAC > ODBA > OTAC > CTAC. The basal spacing variations in DDAC, OTAC, and CTAC positively correlate with their carbon chain length. The phenyl group in ODBA occupies a larger van der Waals volume than the methyl group in OTAC, resulting in greater basal spacing variation for ODBA. Based on simulated results, we propose classifying the substitution process of modifiers in MMT into three stages: filled, saturated, and supersaturated. The substitution amount nodes are transition points between filled and saturated stages across different modifiers. Notably, forming supersaturated modified MMT systems remains challenging under macroscopic experimental conditions at ambient temperature.

### 3.3. Molecular Arrangement and Movement

**Figure 6:**
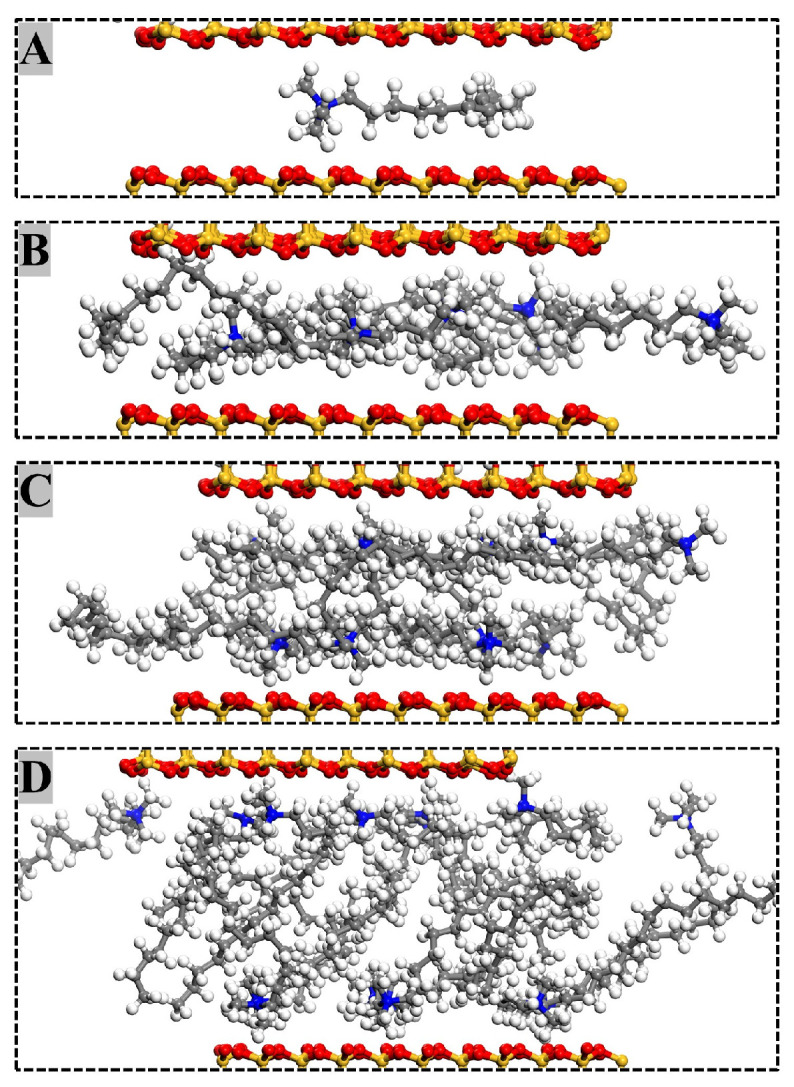
Arrangement of modifiers in MMT interlayers with different substitution amounts: (**A**) OTAC1; (**B**) OTAC6; (**C**) OTAC11; and (**D**) OTAC16.

The arrangement of modifier molecules within MMT interlayers varied with modifier substitution amounts. Figure 6 illustrates the interlamellar arrangement of modifiers in OTAC1, OTAC6, OTAC11, and OTAC16 unit cells within MMT layered structures. In the filled stage (Figure 6A), the substitution amount remained low, allowing modifiers to maintain sufficient mobility between MMT layers for molecular movement within interlayer spaces. With increasing substitution (Figure 6B), modifier molecules underwent lateral expansion to occupy interlayer regions while maintaining minimal variation in MMT layer spacing. In the saturated stage (Figure 6C), the increased substitution amounts exhausted lateral spatial capacity, triggering longitudinal expansion of modifiers that consequently enlarged MMT layer spacing. Concurrently, molecular stratification of modifiers emerged, with N atom segments attracted to upper and lower MMT sheets via charge interactions while terminal regions formed crosslinks. Upon further incorporation of modifier molecules into MMT interlayers (Figure 6D), distinct head–tail segregation manifested. N atom-headed segments maintained specific adsorption distances from MMT layers, while tail regions distal to N atoms underwent crosslinking that structurally separated MMT sheets, leading to basal spacing expansion.

In MD simulations, small molecules with smaller van der Waals volumes (e.g., water) form stratified distributions exhibiting discernible interlayer gaps in MMT interlayers, whereas large molecules with larger volumes (e.g., quaternary ammonium salts) develop continuous packing structures without interfacial separation. Li et al. [32] demonstrated through MD simulations that water molecules adsorbed in MMT interlayer spaces formed two layers. Wang et al. [33] validated this bilayer arrangement, while Zhao et al. [34] observed three water layers. Su et al. [35] investigated the basal spacings and interlayer structures of dodecyl dimethyl benzyl ammonium (DDBA)-MMT. The results showed that increasing interlayer DDBA loadings induced sequential configuration transitions in the interlayer region, corresponding to bilayer, pseudo-trilayer, and pseudo-quadrilayer structural arrangements. The initial basal spacing of MMT in Su’s study was set to 50 Å. Although this spacing sufficiently accommodated organic ions, it still failed to provide adequate movement space for long-chain molecules. Consequently, some modifiers were compressed by others, preventing the positively charged N atom head groups from completing adsorption. Su attributed the emergence of pseudo-four-layer structures to the presence of excessive unattached DDBA in the intermediate regions, which confirmed this viewpoint. Qiu et al. [36] investigated the microstructure evolution of OTAC–MMT complexes, revealing that increasing charge density induces progressive structural transitions from non-uniformly tilted three–four–five layers to six-layer arrangements in the interlayer region. This phenomenon arises because steric hindrance restricts the spatial distribution of subsequently intercalated OTAC molecules. As interlayer OTAC loading increases, later-incorporated molecules become confined to axial regions within the interlayer domain due to limited available space.

Our experiments showed modifier molecules retaining bilayer structures even under complete K^+^ substitution without three-layer transitions. The *c* value of the unit cell in this study was set to 80 Å. After the adsorption was completed through the application of force, the maximum basal spacing of MMT still did not exceed 40 Å. This indicates that the modifier possessed sufficient interlayer space for segmental movement, and the head groups of the modifier completed adsorption prior to the closure of MMT layers. Simultaneously, the high negative charge density of our MMT model may enhance adsorption via stronger electrostatic interactions. Wu [37] similarly observed cetyltrimethylimidazolium chloride’s free movement within MMT layers, consistent with our delamination findings.

### 3.4. Interaction Energy

The interaction energy (*E*_int_) reflects the strength of intermolecular forces [38]. The *E*_int_ between MMT and modifier can be determined using the energy equation [39].

(1) $$E_{\mathrm{int}}=E_{\mathrm{MMT}+\mathrm{QAS}}-(E_{\mathrm{MMT}}+E_{\mathrm{QAS}})$$

where *E*_MMT+QAS_ is the total energy of the unit cell, and *E*_MMT_ and *E*_QAS_ are the individual energies of MMT and modifier. All energies are expressed in units of kcal·mol^−1^.

**Figure 7:**
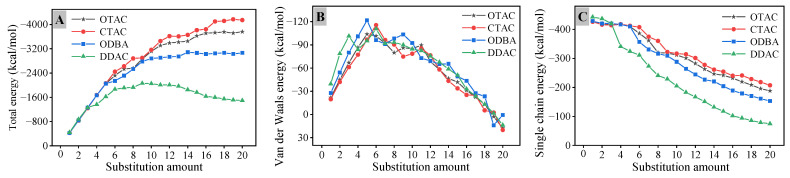
The interaction energy: (**A**) total; (**B**) van der Waals; (**C**) single chain.

The negative value of interaction energy indicates a favorable interaction, and a higher absolute value corresponds to increased interaction strength [40]. As illustrated in Figure 7A, in the filled stage, *E*_int_ exhibits a rapid increase at low substitution amounts resulting from the elevated quantity of modifier molecules. Beyond the node value of substitution amount, occupation of the initial lateral space by modifiers coupled with intensified inter-molecular interactions results in attenuation of *E*_int_ growth rate. When the DDAC substitution amount exceeded 12, significant basal spacing expansion increased the distance between middle chain segments and MMT, while intensified inter-chain segment interactions induced a gradual *E*_int_ reduction with an escalation in the substitution amount. Comparative analysis reveals that the *E*_int_ magnitude follows the order of CTAC > OTAC > ODBA > DDAC at equivalent substitution levels. Shorter modifier chain segments reduce both self-entanglement interactions and basal spacing, thereby promoting closer interaction distances that elevate *E*_int_.

The *E*_int_ is defined as the sum of the electrostatic interaction energy (*E*_elec_) and the van der Waals interaction energy (*E*_vdW_) [41]. In the *E*_int_ of MMT and modifiers, *E*_elec_ constitutes the predominant component, representing more than 90% of the *E*_int_. The maximum *E*_vdW_ contribution originates from DDAC unit cell at 9.00%, whereas other modifiers exhibit *E*_vdW_ contributions ranging from 5.06% to 6.45%. As shown in Figure 7B, the *E*_vdW_ demonstrates three characteristic stages. In the filled stage, *E*_vdW_ increases sharply with substitution quantity due to enhanced molecular proximity. Upon reaching the substitution amount node, the basal spacing expands, causing gradual *E*_vdW_ reduction as van der Waals interactions weaken with increased distance. Under the supersaturated stage, *E*_vdW_ transitions to positive values, reflecting repulsive van der Waals interactions between MMT and the modifier.

The total interaction energy was evenly distributed among individual modifiers to establish the correlation between substitution amount and single chain interaction energy (*E*_single_). As shown in Figure 7C, in the filled stage, increasing substitution amount induces chain segment interference, where *E*_single_ exhibits gradual decline followed by stabilization. Upon reaching the substitution amount node, basal spacing expansion increases the distance between modifier chain segments and MMT, resulting in accelerated *E*_single_ reduction with further substitution. The evolving patterns of various interaction energies collectively validate the three-stage substitution process of modifiers in MMT.

### 3.5. Hydrogen Bond Interactions

H-bond is an electrostatic interaction whose strength exceeds van der Waals interactions but remains weaker than covalent bonds. H-bond fundamentally involves three-body coordination. In the classical X-H—Y configuration, donor (X) and acceptor (Y) atoms are predominantly high-electronegativity nonmetals with compact atomic radii, exemplified by oxygen, fluorine, and nitrogen. These atoms may be either identical or distinct chemical species. This electrostatic interaction specifically arises between partially positive hydrogen (δ + H) and electronegative atomic centers carrying negative charge (δ-Y). H-bond identification in this work followed geometric parameters [42,43], with the X-Y distance constrained to *r* < 3.5 Å. This threshold value corresponds to the first minimum position in the X-Y radial distribution function (RDF).

The RDF quantitatively characterizes material bonding relationships and intermolecular interaction strengths [44]. To investigate MMT–modifier hydrogen bonding, H atoms from the modifier within 4 Å of MMT edges and O atoms in MMT’s outermost layer were analyzed via RDF. The computational formula is expressed as [40]:

(2) $$g_{O\text{-}H}(r)=\frac{1}{4\pi \rho_{H}r^{2}}\times \frac{{\mathrm{dN}}_{O\text{-}H}}{dr}$$

where *ρ*_H_ denotes the number density of selected H atoms, *r* represents the H-O interatomic distance, dN_O-H_ corresponds to the average oxygen atom count within [*r*, *r* + d*r*]. g(*r*) refers to the ratio of the local molecular density at radial distance *r* from a central molecule to the bulk system density; higher g(*r*) values indicate stronger H-O intermolecular interactions.

**Figure 8:**
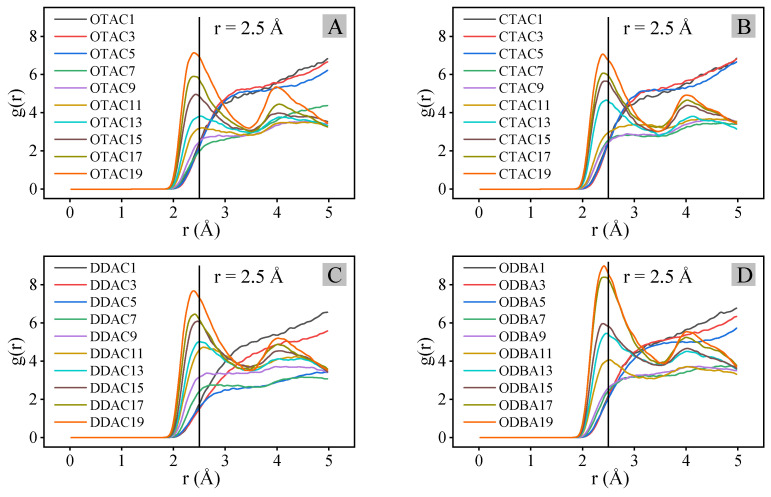
RDF(H-O) analysis curve: (**A**) OTAC; (**B**) CTAC; (**C**) DDAC; and (**D**) ODBA.

**Figure 9:**
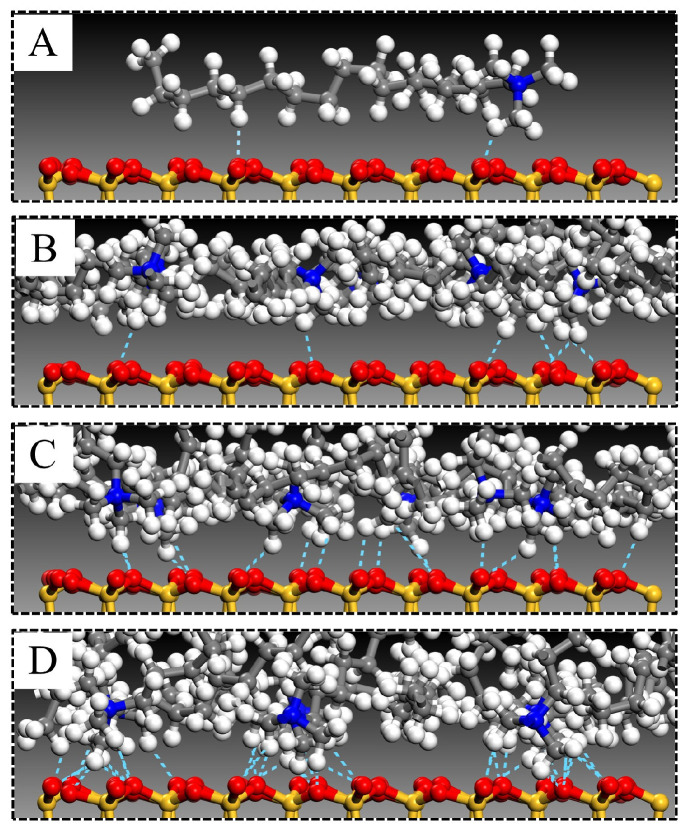
The number of H-bonds in MMT with different substitution levels of modifiers: (**A**) OTAC1; (**B**) OTAC6; (**C**) OTAC11; and (**D**) OTAC16.

Figure 8 presents the RDF (H-O) analysis curves across different modifiers and substitution amounts. In Figure 9, OTAC serves as a representative case where H-bonds are explicitly indicated by light-blue dashed lines. Under the filled stage with low substitution levels, RDF profiles exhibited featureless curves with gradual g(*r*) variations beyond *r* = 3.0 Å, indicating extended H-O separations and minimal hydrogen bonding (Figure 9A). When exceeding the substitution amount node, characteristic peaks emerged at *r* = 2.5 Å (Figure 9B), confirming that H-bond formation is consistent with strong interactions [45]. Progressive substitution increases led to simultaneous peak intensification and *r*-value reduction, demonstrating enhanced H-bond density (Figure 9C,D) and strengthened H-bond strength.

Under the filled and saturated stages, the g(*r*) magnitudes at equivalent substitution quantities followed the hierarchy of DDAC > ODBA > OTAC > CTAC, which is attributed to variations in H-atom density caused by different main-chain C-atom counts among modifiers. Although ODBA and OTAC possess identical main-chain C-atom counts, ODBA exhibits higher H-atom density due to its structural incorporation of benzene ring groups. In the supersaturated stage, ODBA exhibited anomalous RDF profiles with comparatively higher g(r) values than other modifiers, which may originate from simulation artifacts caused by its inherent difficulty in achieving the supersaturated stage under ambient conditions.

### 3.6. Nitrogen Atom Distance

**Figure 10:**
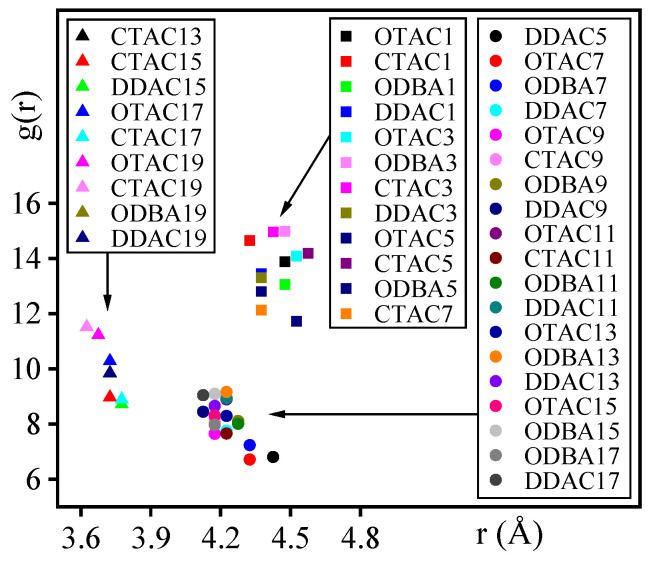
The coordinates of the peak points for each RDF curve.

Throughout the investigation, N-atom distances in modifiers played a critical role during MMT interlayer modification, therefore all N-atoms of modifiers and O-atoms of outermost MMT were subjected to RDF analysis. It should be noted that the limited N-atom population (≤20 in calculations) introduced statistical uncertainty in distance distribution profiles. Quantitative characterization focused on RDF(N-O) peak positions (*x_r_*, *y*_g(*r*)_), where *x_r_* and *y*_g(*r*)_ represent the radial distance and corresponding probability density at peak maxima, respectively, as illustrated in Figure 10.

Figure 10 demonstrates that RDF(N-O) peak positions clustered into three distinct regions across different modifiers and substitution levels, corresponding to the filled, the saturated, and the supersaturated stages, respectively. In the filled stage, N-atoms maintained 4.3–4.6 Å separations from MMT surfaces through charge attraction. In the saturated stage, concurrent charge attraction between N-atoms and MMT layers with intertwined modifier compression established 4.1–4.3 Å distances. The supersaturated stage regime exhibited further distance reduction to 3.6–3.7 Å under intensified compression, where van der Waals repulsion superimposed on charge interactions mechanistically explained the diminished total interaction energy.

### 3.7. Comparison to Experiments

The changes in the basal spacing of OMMT from others’ experimental studies were analyzed and compared with our simulated data to validate its reliability. Qian et al. [19] introduced OTAC into MMT, resulting in a basal spacing expansion from 15.8 Å to 24.7 Å, corresponding to a 56.33% increase. Experimental studies by Li et al. [46] demonstrated that OTAC increased MMT’s basal spacing from 14.4 Å to 2.14 Å, representing a 48.61% enhancement. Shiva et al. [47] and Zhang et al. [48] reported CTAC-induced basal spacing increments of 45.83% and 34.56% in MMT, respectively. Yuan’s research [49] documented an MMT basal spacing of 12.2 Å, with DDAC incorporation at different mass fractions altering OMMT basal distances to 1.38–1.79 Å. Nunes [50] similarly confirmed a 21.85% basal spacing augmentation in MMT through DDAC modification.

**Figure 11:**
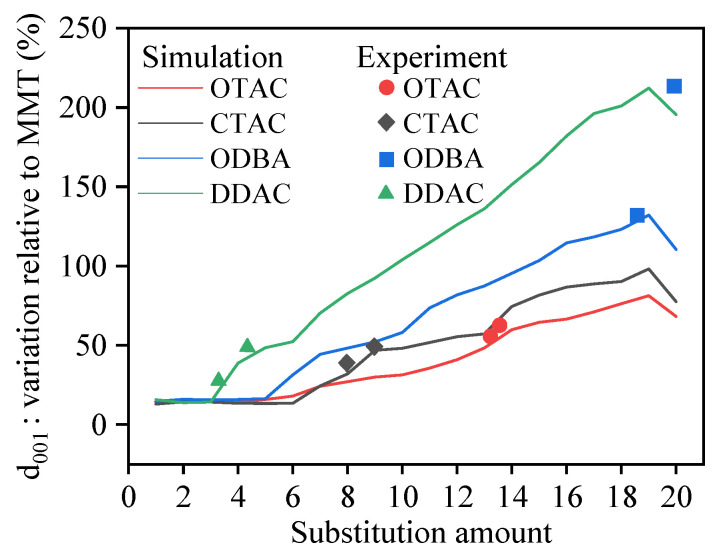
The percentage increase in the d_001_ of MMT in simulation and experiment.

Using the simulated MMT basal spacing of 12.29 Å as the reference, the percentage increases in basal spacing induced by different modifier quantities were calculated, as shown in Figure 11. Experimental results were also annotated in Figure 11. The graph demonstrates that experimental OMMT basal spacing increments predominantly correspond to simulated saturation states, with minority cases matching oversaturation states. Specifically, Jia’s experiment [51] showed ODBA-induced expansion from 12.4 Å to 2.87 Å in MMT, while Gao’s work [52] revealed basal spacing increments from 12.5 Å to 1.47–2.68 Å under varying ODBA concentrations, exceeding maximum simulated values. This discrepancy potentially originates from ODBA’s enhanced ability to separate MMT lamellae in experimental conditions compared to simulation constraints. The computational limitations of the simulation in this study in preventing lamellar separation could be addressed in future studies through aqueous environment implementation and enlarged simulation cell dimensions.

The theoretical findings of this study provide preliminary guidance for determining the dosage of organic modifiers during MMT modification processes. For instance, the Nunes [50] experiment employing 20 g DDAC and 32 g MMT produced OMMT with merely a 0.41 Å increment in basal spacing. However, simulation data reveal substantial potential for further expansion of basal spacing. This implies that increasing DDAC dosage or optimizing synthesis conditions may facilitate the preparation of OMMT with enhanced basal spacing, thereby potentially improving composite material performance.

## 4. Conclusions

This study investigated the effects of four quaternary ammonium organic modifiers on MMT basal spacing through MD simulations. The results demonstrated three characteristic stages during the organic modification process: the filled stage, the saturated stage, and the supersaturated stage, with their underlying mechanisms systematically elucidated at the molecular scale.

During the filled stage, stable basal spacing coincided with rapid *E*_int_ escalation, during which mobile modifiers maintained distant N-atom distances without H-bonds between modifiers and MMT, until reaching critical substitution amounts thresholds (OTAC5, CTAC6, ODBA5, and DDAC3). The saturated stage initiated layer expansion with decelerated *E*_int_ growth, featuring constrained molecular mobility, reduced N-atom distances, and sparse H-bond formation. Transitioning to supersaturation, persistent dilation caused *E*_int_ stabilization or decline through van der Waals repulsion, accompanied by closer N-atom distances and extensive H-bond formation.

The influence of different quaternary ammonium modifiers on MMT basal spacing primarily stems from their steric occupancy. Shorter chains demonstrated reduced steric demands, consequently exhibiting diminished capacity for interlayer expansion. Concurrently, lower chain self-entanglement energy in shorter-chain modifiers resulted in stronger MMT–modifier interfacial interactions. And prolonged MD simulations revealed a bilayer configuration with N atoms adsorbed onto MMT lamellae via electrostatic adsorption, accompanied by self-interpenetrating hydrocarbon tails.

## Figures and Tables

**Table 1:** Basic information on substances.

Substance	Molecular Structure	Model
MMT	\	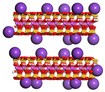
OTAC	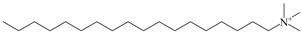	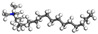
CTAC	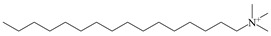	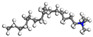
ODBA	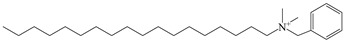	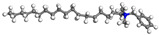
DDAC	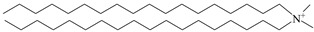	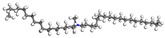

## Data Availability

The original contributions presented in this study are included in the article. Further inquiries can be directed to the corresponding author.

## Abbreviations

The following abbreviations are used in this manuscript:

MMT	Montmorillonite
OMMT	Organic Montmorillonite
MD	Molecular dynamics
OTAC	Octadecyl Trimethyl Ammonium Chloride
CTAC	Cetyl Trimethyl Ammonium Chloride
ODBA	Octadecyl dimethyl benzyl ammonium chloride
DDAC	Dioctadecyl dimethyl ammonium chloride
*E*_int_	Interaction energy
*E*_elec_	Electrostatic interaction energy
*E*_vdW_	Van der Waals interaction energy
*E**_single_*	Single chain interaction energy
H-bond	Hydrogen bond
H	Hydrogen atom
C	Carbon atom
O	Oxygen atom
N	Nitrogen atom
K^+^	Potassium ion
NVT	Canonical ensemble
RDF	Radial distribution function
